# *The Organon* and Materia Medica Club of the Bay Cities of California

**Published:** 1898-04

**Authors:** G. H. Martin

**Affiliations:** Secretary pro tem.


### *THE ORGANON* AND MATERIA MEDICA CLUB OF THE BAY CITIES OF CALIFORNIA.

The regular semi-monthly meeting of *The Organon* and Materia Medica Club convened at the office of Dr. J. M. Selfridge, Oakland, on Friday evening, August 6th, 1897. Members present were Drs. J. M. Selfridge, Augur, Holmgren, G. H. and E. F. Martin. The Secretary being absent, Dr. G. H. Martin acted as Secretary pro tem. The minutes of the annual meeting of July 2d were read and approved. Dr. Augur read an interesting paper on "Syphilis and Sycosis," in which he epitomized Hahnemann's views on the same, as gathered from his several writings.

In the discussion other members gave their understanding of Hahnemann's meaning on certain points, but these did not differ materially, and all agreed that neither science nor time had greatly altered Hahnemann's doctrines concerning these two measures. Adjourned.

G. H. MARTIN,
*Secretary pro tem.*

---

### SYPHILIS AND SYCOSIS.

GEORGE J. AUGUR, M. D.

[Read before *The Organon* and Materia Medica Club of the Bay Cities of California.]

